# Environmental determinants of the occurrence and activity of *Ixodes ricinus* ticks and the prevalence of tick-borne diseases in eastern Poland

**DOI:** 10.1038/s41598-021-95079-3

**Published:** 2021-07-29

**Authors:** Zbigniew Zając, Joanna Kulisz, Katarzyna Bartosik, Aneta Woźniak, Malwina Dzierżak, Adil Khan

**Affiliations:** 1 Chair and Department of Biology and Parasitology, Medical University of Lublin, Radziwiłłowska St. 11, 20-080 Lublin, Poland; 2 District Sanitary and Epidemiological Station in Lublin, Uniwersytecka 12 St., 20-029 Lublin, Poland; 3 Department of Zoology, Abdul Wali Khan University, Mardan, Pakistan

**Keywords:** Ecology, Ecology

## Abstract

*Ixodes ricinus* is the most widely distributed tick species in Europe. Mainly deciduous and mixed forests, pastures, and urban parks are habitats preferred by this species. *I. ricinus* ticks are also one of the most important reservoirs and vectors of human and animal infectious diseases on the continent. *Borrelia burgdorferi* s.l. spirochetes causing Lyme borreliosis (LB) in humans and tick borne encephalitis virus (TBEV), which is a causative agent of tick-borne encephalitis (TBE), are pathogens with the highest medical importance transmitted by this species. Investigations of the environmental determinants of the occurrence and activity of *I. ricinus* are crucial for elucidation of the environmental background of tick-borne diseases. In eastern Poland, *I. ricinus* is a common species with peak activity recorded in May in the entire region. During this period, 49 females, 32 males, and 55 *I. ricinus* nymphs were collected from an area of 900 m^2^. The results of the present study show that the occurrence and seasonal activity of this tick species are mainly influenced by microhabitat conditions, and saturation deficit has a significant effect on the activity of the species. Eastern Poland is characterized by a high incidence of LB and TBE. We have shown a correlation between the forest cover and the number of reported cases of tick-borne diseases.

## Introduction

*Ixodes ricinus* is the most widely distributed species of ticks in Europe^1^, although it is not a dominant species in some regions. For instance, *Dermacentor reticulatus* dominates in eastern Poland, western Ukraine, and the Czech Republic (Moravia)^2–4^. In the Mediterranean countries in the south of Europe, *I. ricinus* co-occurs with numerous populations of ticks from the genera *Hyalomma* and *Rhipicephalus*^5,6^. The geographical distribution range of *I. ricinus* expands from the Iberian Peninsula to the foothills of the Urals and from the Balkan Peninsula to the Arctic Circle in Scandinavia^1,7^. Local island populations of *I. ricinus* have also been described in North African countries, where ticks were collected from vegetation or animals, e.g. Tunisia, Algeria, and Morocco^1,8–10^, and in Asia Minor^11,12^.

*Ixodes ricinus* ticks prefer habitats with mainly deciduous and mixed (less often coniferous) forests with a clear layer of litter. Ticks of this species have also been collected in pastures, moorlands, and urban parks^13–16^. These habitats ensure the optimal level of humidity, especially for juvenile stages, which are most sensitive to water loss^13,17^. Additionally, they are colonized by small rodents, i.e. tick hosts required for completion of the tick life cycle as well as transfer of specimens between habitats and introduction thereof in new areas^18,19^.

*Ixodes ricinus* ticks are among the most important vectors and reservoirs of infectious human and animal diseases in Europe^20^. *Borrelia burgdorferi* s.l. causing Lyme disease (LB) and tick-borne encephalitis virus (TBEV) transmitted by this species to humans are microorganisms with the greatest medical importance^21^. Recently, a systematic increase in the incidence of these diseases has been observed in many European countries. The areas of high risk of tick-borne encephalitis (TBE) include the Baltic republics, southern Scandinavia, eastern Poland, the Czech Republic, Slovakia, Austria, Hungary, Germany, and to a large extent the European part of Russia (where *I. persulcatus* is another TBEV vector)^22,23^. Similarly, most cases of LB on the old continent are reported in Central European countries^22,24^. The geographic range of this disease is wider than that of TBE, as it covers virtually the entire continent^25^.

*Ixodes ricinus* ticks can also transmit rickettsiae *Anaplasma phagocytophilum*, rickettsiae of the spotted fever group *Rickettsia helvetica*, *R. monacenis*, and Candidatus *Neoehrlichia mikurensis*, and protozoa *Babesia* spp. Moreover, the tick species has been found to transmit rare viruses, which may pose difficulties in diagnosis and treatment due to the unknown etiology of the infections that they cause. These include Louping ill virus, Uukuniemi virus, Crimean-Congo hemorrhagic fever virus, Coltivirus Eyach, Orbi virus, and Tribeč virus^26–28^. The presence of genetic material of many other microorganisms, e.g. *Bartonella* spp., has been confirmed in *I. ricinus* ticks as well, but their pathogenicity to humans is still being investigated, or the role of *I. ricinus* bites as a transmission route is limited, as in the case of *Francisella tularensis* and *Coxiella burnetti*^28–30^. In addition to the direct negative effects of *I. ricinus* infestations on human health, treatment of tick-borne diseases and their consequences generates substantial costs in the health care system. The annual cost of treatment of tick-borne diseases in the Netherlands is higher than the cost of 7 out of 14 foodborne pathogens and is estimated in total at € 19.3 million^31^. In turn, in Germany it amounts to approximately € 80 million^32,33^. The high cost of LB treatment is also reported from the United States (estimated at $ 500 million)^34^. In Poland the cost of hospitalization due to LB for one patient is estimated at € 582.39^35^.

The aim of the present study was to investigate the impact of environmental factors on the occurrence and activity of *I. ricinus* ticks in eastern Poland (Lublin Province) and on the prevalence of the most frequently tick-borne diseases diagnosed in humans in this region, i.e. Lyme borreliosis and tick-borne encephalitis.

## Results

### Occurrence and activity of *Ixodes ricinus* ticks

In total, 2375 *I. ricinus* ticks were collected in the study area, including 1086 females, 754 males, and 535 nymphs. The highest density of these ticks was recorded in the central and eastern part of the province (subregions B and C). Up to 49 females and 32 males were collected in these areas during one collection round from an area of 900 m^2^. Nymphs accounted for 22.5% of the general structure of the population in the region, and the largest number of specimens of this stage, i.e. 55 individuals (73.5%), was collected at the site located in Puławy District (Subregion A) in the western part of the region (Table 1). The analyzed subregions did not differ statistically significantly in the size of the local *I. ricinus* populations (H = 3.5688, p = 0.3119), no significant correlation was found between the forest cover and the number of ticks (r = − 0.4057, p = 0.0954) and between the number of wild ungulates (roe deer, deer, moose) and the number of ticks (r = − 0.2722, p = 0.6020).

**Table 1:** Occurrence and number of *Ixodes ricinus* ticks in the study area in 2020 and the proportion of the forest cover in the overall land structure in the analyzed districts (*FC* forest cover, *F* females, *M* males, *N* nymphs).

District/collection site	FC (%)	Months																								Whole study period			Total
		III			IV			V			VI			VII			VIII			IX			X						
		F	M	N	F	M	N	F	M	N	F	M	N	F	M	N	F	M	N	F	M	N	F	M	N	F	M	N	
**Subregion A**																													
Puławy	24.8	0	0	0	4	2	18	6	2	55	8	10	35	0	0	5	0	0	0	4	1	12	5	3	0	27	18	125	170
Opole	29.9	3	0	0	14	3	0	18	12	5	6	8	6	2	4	0	3	7	0	7	2	0	4	4	0	57	40	11	108
Ryki	22.1	0	0	0	6	2	0	8	1	0	2	2	2	2	0	0	0	1	0	0	0	0	0	0	0	18	6	2	26
Kraśnik	21.3	6	3	0	13	9	4	19	17	9	9	9	3	6	2	0	4	8	0	10	14	4	0	0	0	67	62	20	149
**Subregion B**																													
Radzyń	24.9	2	0	0	22	6	4	27	10	5	6	7	0	3	4	0	4	0	0	0	2	0	0	0	0	64	29	9	102
Lublin	10.3	4	8	0	7	8	0	15	13	0	8	2	0	4	0	0	3	7	0	11	8	0	0	2	0	52	48	0	100
Lubartów	20.9	7	8	0	10	8	0	39	21	15	11	15	18	3	2	0	8	0	0	16	14	0	0	3	0	94	71	33	198
Świdnik	11.3	3	1	0	4	8	0	12	6	6	4	1	4	7	3	0	0	0	0	2	0	0	3	0	0	35	19	10	64
Łęczna	13.9	10	2	0	16	13	4	44	20	8	13	13	3	8	5	0	4	4	0	10	4	10	4	8	0	109	69	25	203
Krasnystaw	15.3	9	5	0	10	6	33	38	18	12	13	10	22	9	11	6	2	4	0	4	7	12	4	1	0	89	62	85	236
**Subregion C**																													
Parczew	24.9	7	8	0	18	12	4	49	18	0	14	10	7	10	5	0	3	2	0	0	0	8	0	0	0	101	55	19	175
Włodawa	40.8	1	1	0	4	6	0	11	3	14	5	8	0	0	3	0	0	0	0	3	1	0	0	2	0	24	24	14	62
Hrubieszów	13.1	6	2	0	8	4	15	36	32	18	11	20	14	7	12	0	8	2	0	10	2	8	0	2	0	86	76	55	217
Chełm	18.6	0	3	0	2	4	0	18	19	0	12	22	33	5	2	6	1	7	3	5	0	0	2	6	0	45	63	42	150
**Subregion D**																													
Janów	41.0	5	0	0	8	0	0	9	12	0	8	8	0	3	3	0	4	6	5	6	0	0	0	0	0	43	29	5	77
Biłgoraj	23.5	4	2	0	6	2	0	24	18	3	15	5	8	10	12	0	3	1	0	2	4	3	0	0	0	64	44	14	122
Zamość	23.5	5	0	0	10	3	0	11	2	4	8	0	0	10	3	0	2	0	0	4	1	3	1	0	0	51	9	7	67
Tomaszów	21.9	5	2	0	17	10	11	25	10	33	8	2	15	5	1	0	0	0	0	0	5	0	0	0	0	60	30	59	149
Total		77	45	0	179	106	93	409	234	187	161	152	170	94	72	17	49	49	8	94	65	60	23	31	0	1086	754	535	2375

**Figure 1:**
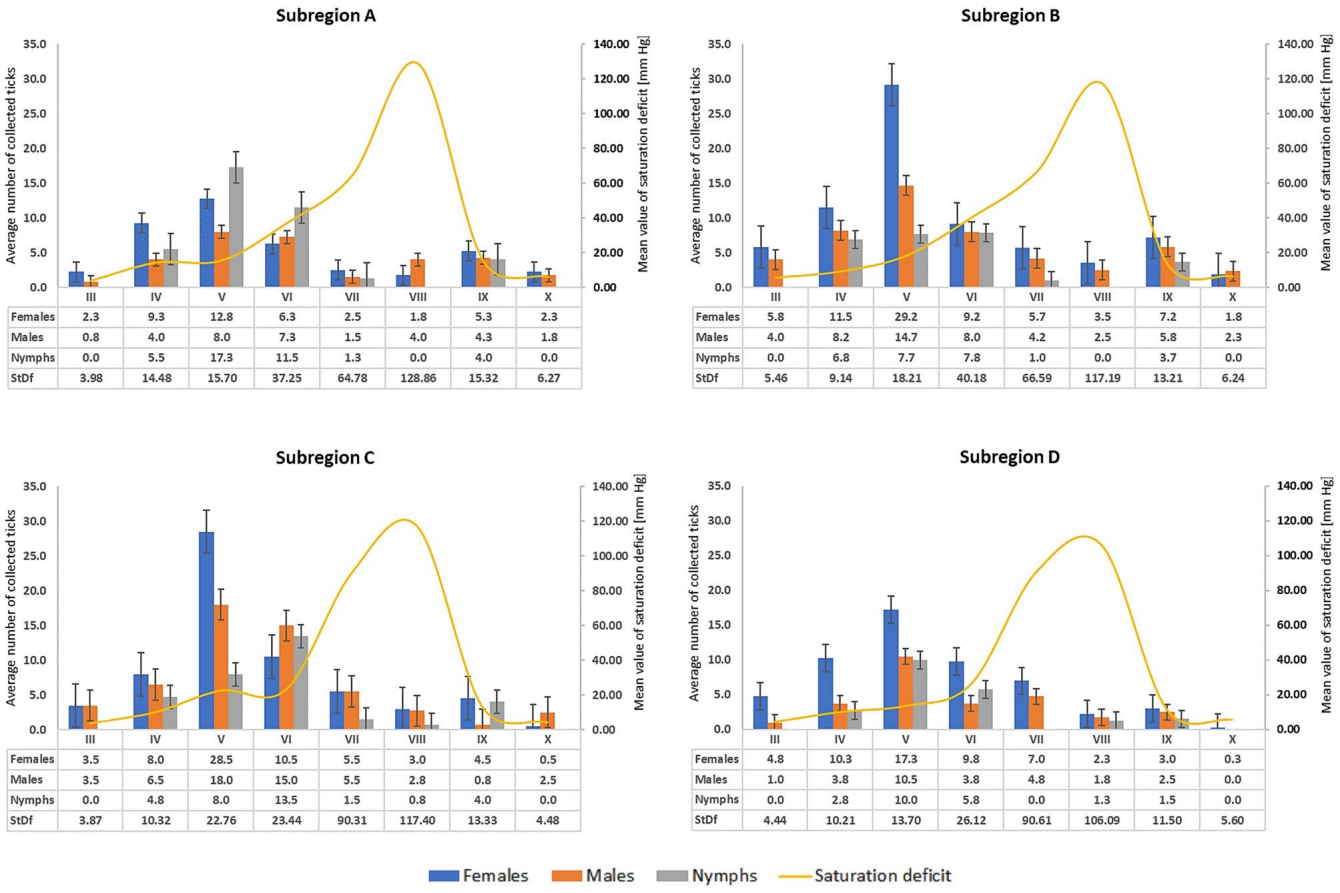
Mean number (with standard error) of *Ixodes ricinus* ticks collected in localities in south-eastern Poland subregions with different microclimatic characteristics (2020).

Distinct rhythms of the seasonal activity of *I. ricinus* were observed in each locality, and its peak (adults) was recorded in May in the study year. In this month, on average 29.2 females and 14.7 males were collected at an average saturation deficit of 13.7–22.76 mmHg in the localities in subregion B. The highest activity of nymphs was noted in May and June. The largest number of nymph specimens was collected in subregion A, which is characterized by the highest average annual air temperatures in the entire region and the longest vegetation season. The activity of nymphs decreased drastically in the summer, when the saturation deficit reached the highest values throughout the observation period (up to 203.44 mmHg). In September, the number of active nymphs was similar to the number of adults, and no activity of this stage was observed in October (Fig. 1, Table 1, Supplementary Table 1). The saturation deficit largely determines the activity of *I. ricinus* (females r = − 0.5633, p = 0.0001; males r = − 0.3428, p = 0.0009; nymphs r = − 0.3611, p = 0.0004).

### Incidence of Lyme borreliosis and tick-borne encephalitis in Lublin Province

**Figure 2:**
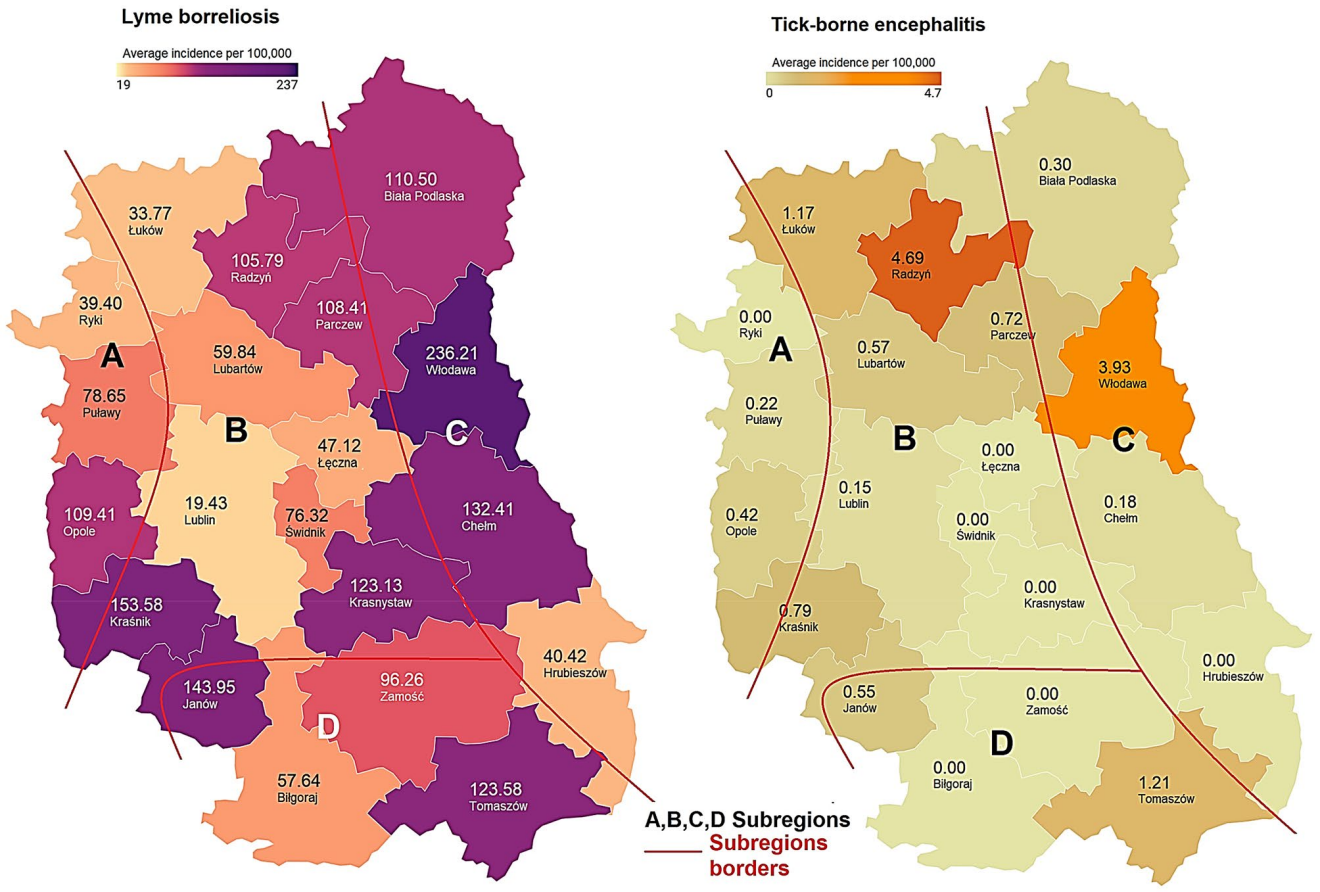
Mean annual incidence of Lyme borreliosis and tick-borne encephalitis per 100,000 inhabitants in the districts of Lublin Province (SE Poland, 2017–2020). Maps were generated in the Datawrapper 1.25 web tool (Datawrapper GmbH, https://app.datawrapper.de/) and modificated in GIMP 2.10 software (GIMP Development Team, https://www.gimp.org/).

In 2017–2020, LB cases were reported from the entire area of Lublin Province. The LB incidence did not differ significantly between the subregions (H = 2.0364, p = 0.5648). The lowest average annual incidence in this period was 19.43 in Lublin District (subregion B), whereas the highest value, i.e. 236.21 cases per 100,000 inhabitants, was recorded in Włodawa District (subregion C) (Fig. 2, Supplementary Table 2). A statistically significant positive correlation was demonstrated between the forest cover and the number of reported LB cases (r = 0.6586, p = 0.0016). There was no correlation between the number of *I. ricinus* ticks and the LB incidence (r = 0.0261, p = 0.9181).

The incidence of TBE in Lublin Province was substantially lower than that of LB. In 2017–2020, the highest number of new TBE infections was recorded in the northeastern districts of Lublin Province (subregion C, partially subregion B). During this period, the highest annual TBE incidence was observed in Włodawa and Radzyń Districts, i.e. 3.93 and 4.69 cases per 100,000, respectively (Fig. 2, Supplementary Table 2). The forest surface area in both districts exceeds the mean value for the province (Table 1). The correlation between the forest cover and TBE incidence was confirmed for the entire province (r = 0.4688, p = 0.0370). In 2017–2020, no case of TBE was reported in six of the 20 districts located mainly in the central and southern part of the province (subregions B, D) (Fig. 2).

## Discussion

*Ixodes ricinus* ticks are the most important reservoirs and vectors of infectious diseases in Europe^20^. Investigations of the habitat determinants of the occurrence and activity of this species in the macro- and micro-regional scale are essential for elucidation of the environmental background of tick-borne diseases.

*Ixodes ricinus* occurs across Europe, but the seasonal activity of the species varies significantly depending on the region. In the temperate maritime climate zone of the western part of the continent, two peaks of activity are usually observed: higher in spring and lower in autumn. In Ireland, ticks of this species are active from February with the peak of activity in adults at the turn of March and April^36^. In Scandinavia, the spring activity peak is noted at the beginning of summer^37^. In southern Germany, the greatest number of *I. ricinus* ticks is collected in May–June^16^^.^ Only one activity peak lasting from June to September is reported in north-western Russia^38^. The results of the present study (Table 1, Fig. 1) show that the activity rhythm of the *I. ricinus* population in eastern Poland is similar to that of the population in a forest area in SW Poland described by Kiewra and Lonc^39^ and ticks reported from Germany^16^, although the period of tick activity during the year is shorter.

The differences in *I. ricinus* activity between the regions in Europe are mainly associated with differences in thermal conditions and ecological habitat types^13^. In the present study, active specimens were collected in a temperature range of 7.5–28.8 °C (14.9–18.5 °C at the peak of activity) (Supplementary Table 1). Temperature is a critical factor inducing tick activity in spring^40^, whereas the humidity level (approx. 70%) has a considerable impact on *I. ricinus* activity during the day/season^41^. The influence of both these factors on the activity of ticks was confirmed in the present study, as the increase in the saturation deficit (integrating the impact of humidity and temperature) was accompanied by a significant reduction in the activity of *I. ricinus* adults and nymphs (Fig. 1). Similar correlations were reported from Italy^42^ and Switzerland^43^.

The density of *I. ricinus* in eastern Poland is one of the highest in the country^44–47^ but lower than that of another ixodid tick species commonly found in this region, i.e. *D. reticulatus*, whose mean density in its preferred habitats is (96.8 specimens/100 m^2^)^2^. In the present study, 5.4 females/100 m^2^ (49 specimens/900 m^2^), 3.5 males/100 m^2^ (32 specimens/900 m^2^), and 6.1 nymphs/100 m^2^ (55 specimens/900 m^2^) of *I. ricinus* were collected during a single collection round (Table 1). No active larvae were collected during the study (Table 1, Fig. 1). This is most probably related to the ecological types of the study plots, which are covered by dense grassland vegetation, while *I. ricinus* larvae are most often collected from the ground or close to rodent burrows^48^.

The subregions examined in the present study did not differ significantly in the number of collected *I. ricinus* specimens. However, the highest density of these ticks in Lublin Province was observed in the central part (subregion B) with a forest cover of 10.3–20.9% and in the eastern part of the region (subregion C) with forests accounting for 13.1–40.8% (Table 1). However, the statistical analysis did not reveal any relationships between the forest cover and the number of ticks. Similarly, no such correlation was reported by Tack et al.^49^. Nevertheless, the authors proved a relationship between the forest margin length and the abundance of *I. ricinus* ticks and indicated the presence of preferred hosts as the main determinant of the tick population size. The presence of rodents, which are often associated with this type of habitat, is particularly important for the development of *I. ricinus*^50^. The present study did not confirm a clear relationship between the number of wild even-toed ungulates (Artiodactyla) and tick abundance. This is most probably associated with the absence of significant differences in the number of these animals between the subregions (Supplementary Table 3). As shown by our observations and literature data, *Capreolus capreolus, Cervus elaphus,* and *Alces alces* living in the study area should be regarded as preferred hosts for adult *I. ricinus* ticks^51,52^.

The present study showed differences in the dynamics of the seasonal activity of *I. ricinus* between the analyzed sub-regions (Fig. 1). In the western part of the province (subregion A characterized by the highest average annual temperature), nymphs were the dominant stage collected at the peak of activity (an average of 17.8 nymphs per one collection round). Subregions B and C were characterized by the highest female activity peak in May, compared with the entire province, while the peak of *I. ricinus* activity in May in subregion D was less numerous and the activity persisted for a longer time (Fig. 1). We believe that these differences result from the local microclimate and microhabitat conditions. Other authors reported a similar relationship as well^53^.

*Borrelia burgdorferi* s.l. spirochetes are one of the most frequent pathogens detected in *I. ricinus* ticks. In Europe, the frequency of this pathogen in *I. ricinus* may vary significantly (depending on the habitat and methodology for detection of genetic material of pathogens) from 0.0 to 61.0%, with an average value of 12.3%^54^. The prevalence of *B. burgdorferi* in the study area in eastern Poland is close to the average value, i.e. 13.1%^55^. Studies conducted in this region showed 1.6% TBEV prevalence in *I. ricinus* ticks^56^. Lublin Province, likewise Podlaskie and Warmińsko-Mazurskie Provinces (north-eastern Poland), has one of the highest values of prevalence of tick-borne diseases in the country^57^. The present results show an average annual LB incidence of 94.79/100,000 inhabitants in Lublin Province in 2017–2020 vs. 52.00/100,000 inhabitants in Poland. Eastern Poland is also an area with an ​​increased TBE risk compared to the rest of the country^57^. Noteworthy, analysis of the number of reported cases of tick-borne diseases should take into account various limitations, e.g. failure to report all cases of TBEV infections, which may be asymptomatic or mildly symptomatic. The lower number of tick-borne diseases (TBDs) reported in 2020 (Supplementary Table 2) is most likely related to the COVID-19 pandemic and may be a result of limited access to health care or reduced mobility of the population. Nevertheless, we believe that the present results based on the long-term average value reflect the actual state of the epidemic in the region.

The forest cover has a considerable impact on the prevalence of LB and TBE in the study area. The highest incidence of tick-borne diseases was observed in districts located in the eastern and northern parts of the province, where the forest cover accounts for 24.9–40.8% (Table 1). Results reported by other authors also indicate that the land cover is the main determinant of LB prevalence in humans^58,59^. Other important factors in the prevalence of TBDs are the density of ticks, in particular nymphs^60^, in the habitat and the presence of rodents^61^. Additionally, the progressing climate change promotes the spread of ticks and transmission of diseases by these arthropods^62^.

The obtained results can be used to develop preventive programs aimed at educating the public on the risk of tick attacks and the transmission of tick-borne diseases in the study area. In addition, these results can be used in planning screening tests for tick-borne pathogens.

## Conclusions

*Ixodes ricinus* ticks occur throughout the entire Lublin Province and their density is one of the highest in Poland. Weather parameters calculated as the value of saturation deficit have a significant influence on the activity of *I. ricinus*. In the study area the relationship between forest cover and the incidence of tick-borne diseases has been observed. Due to the high number of local populations of *I. ricinus* ticks and the high incidence of tick-borne diseases, eastern Poland should be considered as an area of high risk of tick bites and LB and TBE infection.

## Methods

### Study area

The study was conducted in ​​a 20,000-km^2^ area in eastern Poland (Lublin Province) with a population of 2,100,000 residents^63^ (Fig. 1).

**Figure 3:**
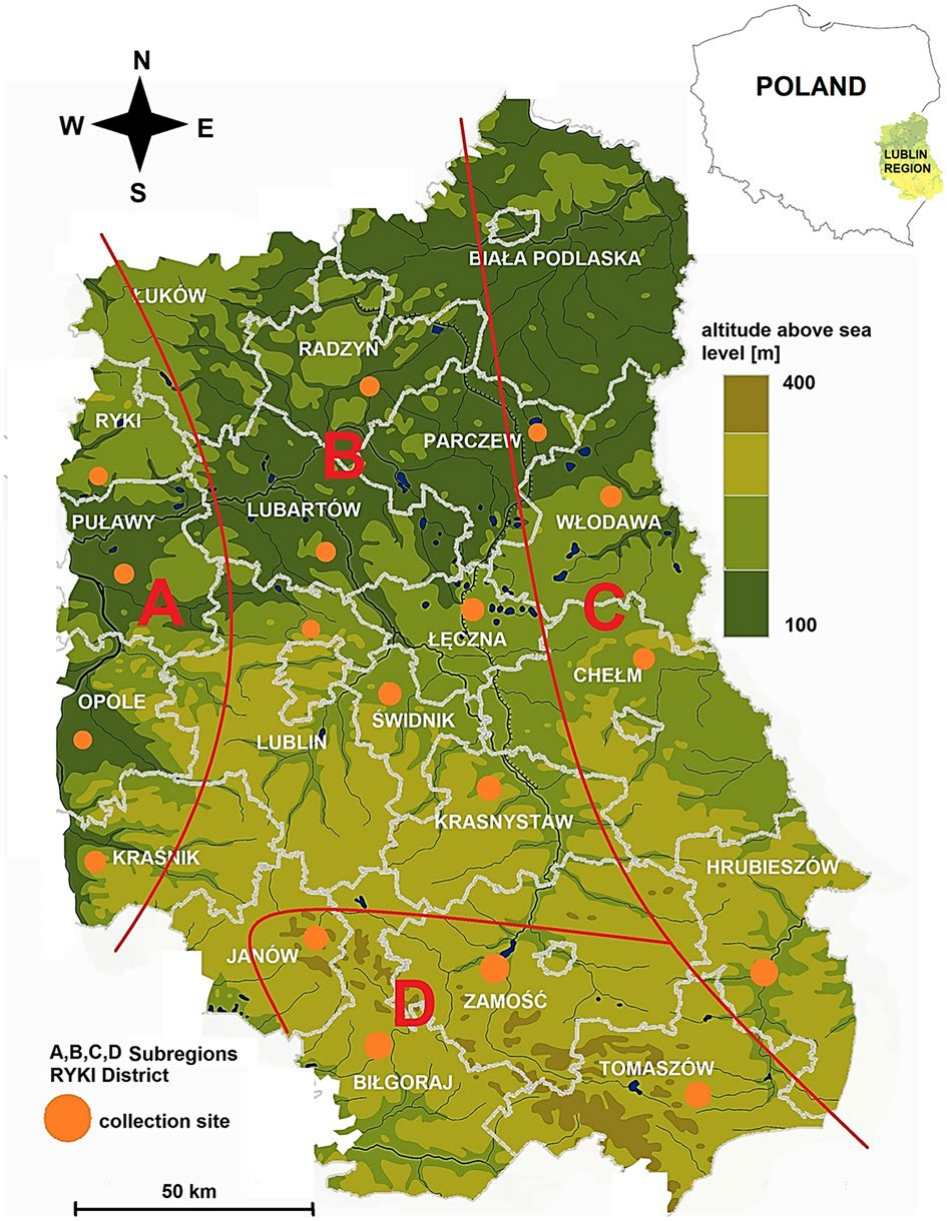
Study area with the division of Lublin Province into subregions with different microclimatic characteristics and marked tick collection sites. Map was generated in ArcGIS 10.0 software (Esri, Redlands, CA, USA, https://www.arcgis.com/) and modificated in GIMP 2.10 software (GIMP Development Team, https://www.gimp.org/).

Based on the division of the Lublin region into climatic areas and taking into account the differences in the length of the growing season^64^, four subregions: A, B, C, and D were distinguished in the area of Lublin Province (Fig. 3).

Subregion A, located along the Vistula River in the western part of the province is characterized by the highest average annual temperature, i.e. 9.4 °C over the last 5 years, and an annual precipitation sum of 650 mm^64,65^. Arable fields and orchards associated with the mild climate dominate in the land use structure. The forest structure is dominated by mixed forests^63^. The potential vegetation represents the Potentillo albae-Quercetum, Tilio-Carpinetum, and island Querco-Pinetum^66^.

Subregion B, covering the central part of the Lublin region, is characterized by an average annual air temperature of 9.2 °C and the lowest annual precipitation in the region i.e. 500 mm^64,65^. With its fertile soils and the relatively long growing season exceeding 200 days^61^ (especially in the southern part of the subregion), the area is intensively used for agriculture. The proportion of the forest cover is the lowest in this area, as it accounts for 16.1%^63^. Tilio-Carpinetum is the dominant of potential vegetation^66^.

Subregion C in the eastern part of the province has a climate with continentalism features, i.e. cold winters and hot summers. The average annual air temperature is 9.2 °C, the growing season below 200 days is the shortest in the entire region, and the annual precipitation sum is 550 mm^64,65^. This area is characterized by a relatively high proportion of agricultural wasteland in the overall land structure and a high forest cover index of 40.8%^63^. The potential vegetation represents the Querco-Pinetum, Carici elongatae-Alnetum, Peucedano-Pinetum and Tilio-Carpinetum^66^.

Subregion D covers the area of Roztocze, i.e. a physico-geographical region characterized by the highest altitudes in the region above 300 m.a.s.l. The average annual air temperature is 9.3  C, and the annual precipitation sum exceeding 700 mm is the highest value in the entire province^64,65^. The landscape structure is dominated by a mosaic of small arable fields often separated by mid-field scrubs. This subregion has a high forest cover index of 40.0%, especially in the eastern part^63^. In contrast to the other subregions, the tree stand is dominated by beech (*Fagus sylvatica*), fir (*Abies alba*), and spruce (*Picea abies*). The potential vegetation is classified as Leucobryo-Pinetum, Querco-Pinetum, and Dentario glandulosae-Fagetum^66^.

### Tick surveillance

The tick collection sites were designated within the administrative borders of the counties of Lublin Province (with the exception of Biała Podlaska and Łuków Districts) following the one district-one site principle and taking into account the division into subregions A-D described above (Fig. 3). In total, 18 sites were selected. Ticks were collected from mid-forest clearings surrounded by deciduous trees. Before starting the collection, the plots were checked for the presence of ticks. A 900-m^2^ (30 × 30 m) plot was established in each site. Ticks were collected for 40 min.

The ticks were collected during the vegetation season at monthly intervals (usually every third week of the month) from March to October 2020. The standard flagging method was employed. A 1-m^2^ flannel cloth was used to sweep the vegetation in the plots. Each time after covering ca. 15 m, the cloth was turned over and inspected to detect the presence of ticks attached to the fabric. The specimens collected in this way were transferred with metal tweezers into a 100-cm^3^ plastic container containing a moist cotton swab to provide approx. 75% relative humidity. Next, the ticks were transported to the laboratory to identify the species and developmental stage of the specimens using a Zeiss STEMI DV4 stereoscopic microscope (Carl Zeiss Light Microscopy, Göttingen, Germany) and a tick identification key^7^.

### Impact of weather conditions on *Ixodes ricinus* activity

In each tick collection round, the current weather conditions, i.e. temperature and relative air humidity, were measured using the Data Loger R6030 device (Reed Instruments, Wilmington, NC, USA). The data were used to calculate the saturation deficit coefficient with the following formula according to Randolph and Storey^67^:

$$StDf=\left(1-\frac{RH}{100}\right)\times {4.9463\times \mathrm{e}}^{0.0621\times \mathrm{T}},$$

where StDf is the saturation deficit; RH is the relative humidity; e is the actual vapor pressure; T is the temperature.

### Prevalence of LB and TBE infection in humans in the Lublin region

Data on the number of reported cases of Lyme disease and tick-borne encephalitis in Lublin Province with division into districts were provided by the Provincial Sanitary and Epidemiological Station in Lublin.

### Forest cover and number of wild animals

Data on the forest cover in the study area were obtained from the information bulletin of the Statistical Office in Lublin^63^. Data on the number of wild animals living in the study area were obtained from the Research Station of the Polish Hunting Association in Czempiń (www.czempin.pzlow.pl).

### Statistical analysis

The normality of the distribution of the data was checked using the Shapiro–Wilk test. Differences in the number of ticks and reported cases of TBDs between the subregions were tested using ANOVA on ranks test. The effect of saturation deficit on tick activity was analyzed with rho-Spearman correlation. The relationships between the forest cover and the number of ticks, the number of wild animals and the number of ticks, the forest cover and the number of reported LB and TBE cases, and the number of ticks and the incidence of tick-borne diseases (LB and TBE) were analyzed with rho-Spearman correlation.

A level of significance of *p* ≤ 0.05 was assumed in all statistical tests. The statistical analysis was conducted using Statistica 10PL (StatSoft, TIBCO Software Inc., Palo Alto, CA, USA) software.

## Supplementary Information

Supplementary Tables.
