# Supplementary material for: Environmental determinants of the occurrence and activity of Ixodes ricinus ticks and the prevalence of tick-borne diseases in eastern Poland
Source: Sci Rep. 2021 Jul 29;11:15472. doi: 10.1038/s41598-021-95079-3 (PMC8322139; doi:10.1038/s41598-021-95079-3)
Supplement: Supplementary file 1 — Supplementary Tables. [file 41598_2021_95079_MOESM1_ESM.pdf]

District	Month																							
	III			IV			V			VI			VII			VIII			IX			X		
	**T**	**RH**	**StDf**	**T**	**RH**	**StDf**	**T**	**RH**	**StDf**	**T**	**RH**	**StDf**	**T**	**RH**	**StDf**	**T**	**RH**	**StDf**	**T**	**RH**	**StDf**	**T**	**RH**	**StDf**
	[ºC]	[%]	[mm Hg]	[ºC]	[%]	[mm Hg]	[ºC]	[%]	[mm Hg]	[ºC]	[%]	[mm Hg]	[ºC]	[%]	[mm Hg]	[ºC]	[%]	[mm Hg]	[ºC]	[%]	[mm Hg]	[ºC]	[%]	[mm Hg]
Subregion A																								
Puławy	9.9	77.0	3.78	19.0	85.5	16.25	17.0	70.0	17.19	19.0	63.7	28.84	24.2	60.1	76.27	24.9	55.5	117.98	13.8	60.6	17.22	13.0	71.0	7.68
Opole	13.0	72.5	7.41	17.7	73.3	18.72	17.6	69.0	19.80	18.7	55.1	28.16	22.9	59.0	50.98	25.1	50.8	119.34	15.8	66.7	14.18	12.5	75.4	6.24
Ryki	8.0	88.2	1.55	16.8	72.1	15.76	14.9	77.0	9.38	20.5	55.6	41.44	23.6	50.9	49.82	24.8	45.0	102.01	16.0	53.7	16.53	12.2	80.9	4.83
Kraśnik	9.7	80.5	3.18	12.9	72.8	7.19	17.0	72.3	16.43	21.6	67.1	50.57	25.0	54.2	82.07	26.8	47.7	176.13	15.0	59.0	13.36	13.0	77.7	6.34
Subregion B																								
Radzyń	7.5	70.0	3.27	17.0	80.2	13.13	18.5	71.0	23.28	21.0	68.2	43.31	25.0	44.9	61.18	23.0	58.7	74.38	15.5	63.6	13.93	11.5	77.7	4.82
Lublin	11.0	81.0	3.89	15.1	77.7	9.54	17.3	72.0	17.60	22.3	54.9	62.45	26.1	47.7	91.83	27.0	51.4	194.34	14.3	63.0	11.17	14.0	69.3	9.59
Lubartów	12.3	81.1	4.89	15.0	61.0	13.04	17.0	71.0	16.86	17.8	66.0	21.70	23.7	44.4	41.83	23.9	45.0	82.71	16.2	67.0	15.35	8.9	83.5	2.42
Świdnik	9.0	69.9	4.06	14.7	65.8	11.64	15.9	74.0	12.55	19.9	57.1	36.11	22.2	50.0	33.19	25.9	51.0	146.14	15.0	60.0	13.20	13.6	70.9	8.57
Łęczna	12.0	74.0	5.93	16.0	80.0	10.60	18.4	62.0	25.66	21.0	61.9	45.70	24.4	55.6	71.37	24.0	43.2	82.41	13.9	60.0	10.82	12.4	72.7	6.59
Krasnystaw	10.0	77.8	3.73	11.9	66.8	6.90	15.5	67.0	13.31	19.4	62.2	31.84	25.0	60.5	97.17	25.1	55.0	123.18	15.7	61.5	14.83	11.5	73.7	5.50
Subregion C																								
Parczew	11.0	74.3	4.95	17.0	70.0	17.19	15.9	77.3	11.39	19.0	57.8	29.84	23.0	53.9	46.12	25.9	61.0	155.0	16.2	51.0	17.22	11.1	80.5	4.04
Włodawa	10.4	80.0	3.66	15.0	75.5	10.01	17.0	80.6	12.86	18.2	77.0	18.99	25.1	68.8	123.04	24.0	70.0	89.73	16.0	70.2	13.93	10.8	80.3	3.86
Hrubieszów	10.5	71.0	5.00	14.5	81.1	7.47	17.0	67.0	18.01	18.3	70.6	22.55	25.0	52.0	76.50	24.8	48.8	108.34	14.9	59.0	13.12	13.4	78.0	6.75
Chełm	8.8	80.5	1.88	12.1	70.5	6.61	15.8	77.0	48.80	18.0	66.7	22.38	26.1	55.0	115.57	25.0	51.2	116.55	12.6	55.1	9.05	9.8	71.0	4.46
Subregion D																								
Janów	11.1	70.0	5.63	15.0	71.9	10.99	16.1	65.0	15.42	19.5	59.3	32.92	24.6	48.7	62.14	24.8	47.0	105.58	13.8	60.5	10.56	11.0	80.5	3.97
Biłgoraj	12.1	77.6	5.39	14.3	70.6	9.84	16.0	73.0	13.13	18.5	66.6	25.05	23.8	45.7	45.15	26.1	40.6	129.14	14.5	55.5	12.48	11.3	77.0	4.78
Zamość	10.0	82.0	3.13	15.8	80.0	10.22	17.8	80.1	15.67	18.2	71.9	21.41	26.6	70.5	203.44	24.1	60.0	96.95	15.5	77.3	10.48	11.9	82.9	4.17
Tomaszów	9.0	73.9	3.63	14.3	70.9	9.80	14.9	72.8	10.57	18.7	69.2	25.09	22.7	62.4	51.73	23.9	60.5	92.70	14.7	60.0	12.48	14.0	69.8	9.48

Supplementary Tab. 1. Weather conditions during collection of *Ixodes ricinus* ticks (T – temperature, RH – relative humidity, StDf – saturation deficit)

District	Population	Number of cases								Average number of cases ± SD 2017-2020		Average incidence per 100,000 ± SD 2017-2020	
		2017		2018		2019		2020					
		LB	TBE	LB	TBE	LB	TBE	LB	TBE	LB	TBE	LB	TBE
Subregion A													
Puławy	112835	151	1	110	0	86	0	8	0	88.75 ± 52.09	0.25 ± 0.50	78.65 ± 53.30	0.72 ± 0.51
Opole	58952	100	1	65	0	65	0	28	0	64.50 ± 25.46	0.25 ± 0.50	109.41 ± 49.87	0.42 ± 0.97
Ryki	55841	42	0	23	0	21	0	2	0	22.00 ±14.15	0.00 ± 0.00	39.40 ± 29.28	0.00 ± 0.00
Kraśnik	95064	146	0	209	0	173	3	56	0	146.00 ± 56.56	0.75 ± 1.50	153.58 ± 68.70	0.79 ± 1.82
Łuków	106591	53	2	46	1	38	2	7	0	36.00 ± 17.56	1.25 ± 0.95	33.77 ± 19.02	1.17 ± 0.54
Subregion B													
Radzyń	58604	68	3	58	2	79	3	43	3	62.00 ± 13.24	2.75 ± 0.50	105.79 ± 26.10	4.69 ± 0.98
Lublin	495477	62	0	149	1	150	1	24	1	96.25 ± 54.91	0.75 ± 0.50	19.43 ±12.79	0.15 ± 0.11
Lubartów	88152	109	0	68	0	27	2	7	0	52.75 ± 39.21	0.50 ± 1.00	59.84 ± 51.37	0.57 ± 1.31
Świdnik	71739	28	0	54	0	92	0	45	0	54.75 ± 23.44	0.00 ± 0.00	76.32 ± 37.73	0.00 ± 0.00
Łęczna	57298	27	0	37	0	17	0	27	0	27.00 ± 7.07	0.00 ± 0.00	47.12 ± 14.25	0.00 ± 0.00
Krasnystaw	62940	68	0	79	0	99	0	64	0	77.50 ± 13.57	0.00 ± 0.00	123.13 ± 24.90	0.00 ± 0.00
Subregion C													
Parczew	34590	45	0	42	1	40	0	23	0	37.50 ± 8.55	0.25 ± 0.50	108.41 ± 28.57	0.72 ± 1.66
Włodawa	38207	99	1	127	2	98	2	37	1	90.25 ± 32.97	1.50 ± 0.57	236.21 ± 99.35	3.93 ± 1.51
Hrubieszów	62473	19	0	25	0	44	0	13	0	25.25 ± 11.62	0.00 ± 0.00	40.42 ± 21.49	0.00 ± 0.00
Chełm	139340	202	0	208	0	201	1	127	0	184.50 ± 33.30	0.25 ± 0.50	132.41 ± 27.60	0.18 ± 0.41
Biała	167648	244	0	246	0	229	1	22	1	185.25 ± 94.48	0.50 ± 0.57	110.50 ± 65.07	0.30 ± 0.34
Subregion D													
Janów	45503	70	0	68	0	74	1	50	0	65.50 ± 9.20	0.25 ± 0.50	143.95 ± 23.36	0.55 ± 1.26
Biłgoraj	100617	141	0	27	0	48	0	16	0	58.00 ± 49.27	0.00 ± 0.00	57.64 ± 56.55	0.00 ± 0.00
Zamość	169072	205	0	213	0	135	0	98	0	162.75 ± 48.14	0.00 ± 0.00	96.26 ± 32.88	0.00 ± 0.00
Tomaszów	82337	96	1	97	3	127	0	87	0	101.75 ± 15.08	1.00 ± 1.21	123.58 ± 21.16	1.21 ± 1.85

Supplementary Tab. 2. Number of cases and the average incidence of LB and TBE in districts of the Lublin Province in 2017-2020 (LB – Lyme borreliosis, TBE – tick borne encephalitis, SD – standard deviation)

Gatunek	Hunting Zone (Districts of Lublin Privnce)					
	**Biała** (Parczew, Biała)	**Chełm** (Chełm, Włodawa)	**Lublin** (Lublin, Lubartów, Łęczna, Opole, Świdnik, Krasnystaw, Puławy)	**Siedlce** (Radzyń, Ryki, Łuków)	**Zamość** (Zamość, Tomaszów, Hrubieszów, Biłgoraj)	**Tarnobrzeg** (Janów, Kraśnik)
Alces alces	1271	1211	1228	1309	705	361
Cervus elaphus	2751	2327	1606	1658	1941	2479
Capreolus capreolus	12187	10946	15457	18853	16708	10361

Supplementary Tab. 3. Estimated number of selected even-toed ungulates (Artiodactyla) in hunting zones, based on www.czempin.pzlow.pl
